# HIF1-alpha overexpression indicates a good prognosis in early stage squamous cell carcinomas of the oral floor

**DOI:** 10.1186/1471-2407-5-84

**Published:** 2005-07-21

**Authors:** Thomas Fillies, Richard Werkmeister, Paul J van Diest, Burkhard Brandt, Ulrich Joos, Horst Buerger

**Affiliations:** 1 Department of Cranio-Maxillofacial Surgery, University of Münster, Waldeyerstrasse 30, 48129 Münster, Germany; 2 Department of Oral and Maxillofacial Surgery, Central German Armed Forces Hospital, Rübenacher Str. 170, 56072 Koblenz, Germany; 3 Department of Pathology, University Medical Center Utrecht, Utrecht, The Netherlands; 4 Institute of Clinical Chemistry and Laboratory Medicine, University of Muenster, Albert-Schweizer-Str. 29, 48129 Münster, Germany; 5 Institute of Pathology, University of Muenster, Domagkstraße 17, 48149 Münster, Germany

## Abstract

**Background:**

Hypoxia-inducible factor 1 (HIF-1) is a transcription factor, which plays a central role in biologic processes under hypoxic conditions, especially concerning tumour angiogenesis. HIF-1α is the relevant, oxygen-dependent subunit and its overexpression has been associated with a poor prognosis in a variety of malignant tumours. Therefore, HIF-1α expression in early stage oral carcinomas was evaluated in relation to established clinico-pathological features in order to determine its value as a prognostic marker.

**Methods:**

85 patients with histologically proven surgically treated T1/2 squamous cell carcinoma (SCC) of the oral floor were eligible for the study. Tumor specimens were investigated by means of tissue micro arrays (TMAs) and immunohistochemistry for the expression of HIF-1. Correlations between clinical features and the expression of HIF-1 were evaluated by Kaplan-Meier curves, log-rank tests and multivariate Cox regression analysis.

**Results:**

HIF-1α was frequently overexpressed in a probably non-hypoxia related fashion. The expression of HIF-1α was related with a significantly improved 5-year survival rate (p < 0.01) and a significantly increased disease free period (p = 0.01) independent from nodal status and tumour size. In primary node negative T1/T2 SCC of the oral floor, absence of HIF-1α expression specified a subgroup of high-risk patients (p < 0.05).

**Conclusion:**

HIF-1α overexpression is an indicator of favourable prognosis in T1 and T2 SCC of the oral floor. Node negative patients lacking HIF-1α expression may therefore be considered for adjuvant radiotherapy.

## Background

Numerous molecular-biological and clinico-pathological studies have increased the knowledge about the hypoxia inducible regulation system in tumour biology. Protection against hypoxia in solid tumours is an important step in tumour development and progression. One system in hypoxia protection of tumour cells is represented by the hypoxia-inducible factor 1 (HIF-1) system which plays a crucial role in biologic processes under hypoxic conditions, especially in angiogenesis and carcinogenesis. HIF-1 is a heterodimeric protein consisting of an alpha-and beta subunit [1], in which the HIF-1α subunit mediates HIF-1 function as a transcription factor in response to cellular hypoxia. Being stabilized under decreased tissue oxygen concentration, it works as a cellular oxygen-sensing system, and induces the activation of key regulations systems through more than 40 proteins, including members of the glucose transporter (GLUT) and carbonic anhydrase (CA) family in the respective tumour cells [2]. Alteration and overexpression of HIF-1α has been detected in a variety of solid tumours, including breast, lung, ovarian and oral cancer with varying (diffuse and perinecrotic) staining patterns [3-7]. The expression of HIF-1α turned out to be of prognostic relevance in different tumours reviewed by Semenza [2]. The prognostic relevance of HIF-1α in tumours derived from squamous epithelium is however controversial.

Independently of improved operation-and adjuvant therapy strategies, the cure rate of oral squamous carcinoma has not changed significantly over the last decades. It still remains at approximately 50% over-all survival [8,9]. Better understanding of the biology of squamous cell carcinoma (SCC) of the oral cavity could identify new prognostic and predictive factors allowing tailoring of surgical and adjuvant therapy

In view of its important role in various cancers, we evaluated HIF-1α expression and related proteins such as GLUT 1 and CA IX in SCC of the oral cavity, which has not well been documented. The results were correlated with clinico-pathological features and prognosis. Our results demonstrate that non-hypoxia driven HIF-1α-expression is a frequent finding in SCC of the oral cavity and is associated with a favourable prognosis.

## Methods

### Patients and tumour material

Patients with histologically proven squamous cell carcinoma of the oral floor treated surgically were eligible for the study. Surgical treatment included radical tumour resection of the whole tumour with a free histopathological margin of at least 4 mm from the tumour borders. Selective neck dissection of Level I, II, III and V was performed in case of suspect results in preoperative tumour staging by computerized tomography or sonography examination or in case of tumour size over 2 cm. Bilateral selective neck dissection was performed when the tumour was extending over the midline (according to the recommendation of Robins et al. 2002)[10]. Radiotherapy was given when lymph node metastases were detected histologically. The series comprised 85 patients (71 men and 14 women) with a median age of 57 years (range 33–87). All tumours were classified postsurgically according to the TNM system (2002) [11]. Patients were clinically evaluated in our routine follow-up.

### Immunohistochemistry

For tissue microarray (TMA) construction, two punch biopsies with a diameter of 0.6 mm were taken from the centre and the periphery (tumour stromal interface) of each tumour and transferred into the new acceptor block. TMA construction was performed by using a special tissue microarray instrument (Beecher Instruments, New Jersey, USA), according to standard protocols [12,13].

Immunohistochemistry for HIF-1 alpha, its downstream genes CAIX and Glut-1, and Ki67 was performed on 4-μm thick slides from the TMA. After deparaffinization and rehydration, endogenous peroxidase activity was blocked for 30 minutes in methanol containing 0.3% hydrogen peroxide. After antigen retrieval, a cooling-off period of 20 minutes preceded the incubation of the primary antibody (anti-HIF-1α; 1/500 dilution; BD Transduction Laboratories, Lexington, Kentucky, USA). Thereafter, the catalyzed signal amplification system (DAKO, Glostrup, Denmark) was used for HIF-1 alpha staining according to the manufacturer's instructions. The antibodies were detected by a standard avidin-biotin complex method with a biotinylated rabbit anti-mouse antibody (DAKO) and an avidin-biotin complex (DAKO), and developed with diaminobenzidine. Before the slides were mounted, all sections were counterstained for 45 seconds with hematoxylin and dehydrated in alcohol and xylene. Appropriate negative (obtained by omission of the primary antibody) and positive controls were used throughout.

For CA IX staining, sections were incubated without antigen retrieval with mouse anti-CA IX (MN 75; 1/50 dilution) for 30 minutes at 20°C and subsequently developed with an avidin-biotin-peroxidase complex method (Envision system peroxidase mouse; Dako). All sections were developed using diaminobenzidine, and subsequently counterstained with haematoxylin.

For GLUT-1 staining, sections were incubated without antigen retrieval with a rabbit polyclonal anti-GLUT-1 antibody (clone A 3536; Dako) and subsequently developed with a standard avidin-biotin-peroxidase complex method (biotinlyated goat antirabbit antibody; Dako; streptavidin peroxidase system; Dako) using an autostainer (Autostainer 480-2D; LabVision, Freemont, California, USA).

For Ki-67 staining (anti Ki-67, Mouse MAb, Immunotech SA, Marseille, France), sections were incubated with mouse anti-Ki-67 (MN 75; 1/40 dilution) over night at 4°C and subsequently developed with an avidin-biotin-peroxidase complex method (Envision system peroxidase mouse; Dako).

For Cyclin D1 staining (anti cyclin D1, Mouse MAb, Santa Cruz Biotechnology, Santa Cruz, USA), sections were incubated with mouse anti-cyclin D1 (1/400 dilution) for 20 minutes at 20°C and subsequently developed with an avidin-biotin-peroxidase complex method (Envision system peroxidase mouse; Dako).

The expression of HIF-1α was determined independently by two pathologists (PJ.v.D., H.B.). Both pathologists determined the percentage of positive cell nuclei in each core. The mean percentage value of the two cores representing one tumour were used for further evaluation. Three different cut offs for overexpression were set at 1%, 5% and 10% of positively stained nuclei.

### Statistical analysis

TNM-stage, histological differentiation and expression of HIF-1α were related to the duration of the progression-free and the overall survival. The measurement of time started from the date of surgery to the date of histologically proven recurrent or metastatic carcinoma or disease related death, respectively. Patients who died from intercurrent diseases were censored at the date of death. Patients lost to follow-up were censored at the date of the last examination. Progression-free survival curves and the overall survival curves were constructed according to Kaplan and Meier [14]. The log-rank test was used to assess differences between groups and the multivariate survival analysis was performed with Cox regression [15].

Correlations between clinicopathologic features and expression of HIF-1α were evaluated by chi-square test. A p-value < 0.05 was considered to be significant.

## Results

Clinical and tumour details of the 85 patients with oral floor squamous cell cancer are shown in Table 1.

HIF-1α expression was confined to the nuclei of neoplastic cells (according to Beasley et al. [4] and Bos et al. [16]). The expression levels did not differ much for the vast majority of the cases between the two tumour cores representing one case, indicating a rather homogeneous expression throughout the tumours. The difference between the evaluations of %HIF-1α expressing cells by both pathologists was within the range of 10%.

Detectable levels of HIF-1α (HIF-1α ≥ 1%) were found within the tumour cells in 63,5% (54/85) of the oral SCCs. Low levels of nuclear staining (HIF-1α ≥ 1% and < 5%) were found in 11,6 % (9/85) of the tumour specimen with detectable HIF-1α-expression, high levels of HIF 1-α expression (≥ 5%) were found in 52,9% (45/85) of the positive tumour specimens and very high levels (≥10%) were found in 35,9% (30/85) of the cases. HIF-1α expression was usually diffuse and tended to be more prominent towards the centre of tumour fields, sometimes associated with a better differentiation, indicated by the presence of keratinized tumour parts. In less than 5% of the cases necrotic areas were seen, and expression of HIF-1α could also be detected in perinecrotic vital tumour cells. HIF-1α-expression could be detected in the tumour stroma in only 5 cases. Cytoplasmatic expression was in 7 of 45 cases, all associated with nuclear staining levels of HIF-1α ≥ 5 %. 89.5 % (76/85) of the specimens showed membrane expression of Glut1 and 26% (12/85) of CA IX of neoplastic cells. No correlation could be demonstrated between HIF-1α, CA IX or Glut I, respectively.

No correlation was found between tumour size, tumour differentiation, lymph node status and expression of HIF-1α (≥1%, ≥5%, ≥10%). Also no correlation between the three expression levels of HIF-1α, cyclin D1 and Ki-67 could be shown in Chi-square test. Kaplan-Meier analysis of HIF-1α expression ≥1% and ≥10% showed no significantly association with disease free (p > 0.05) and overall survival (p > 05).

**Figure 1:**
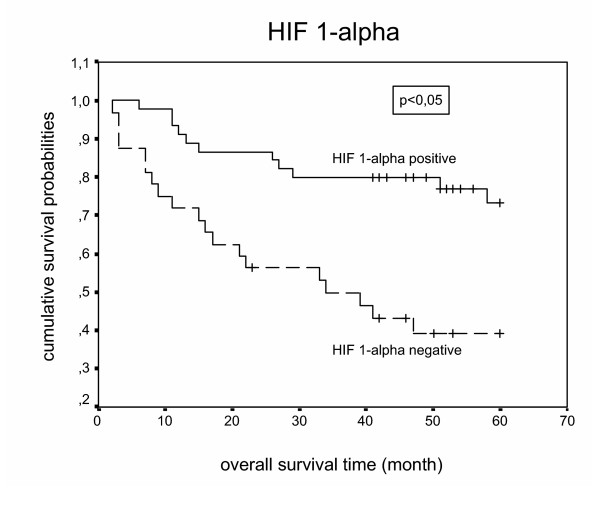
Overall survival of patient with oral squamous cell carcinoma in dependence on HIF-1 expression calculated by the Kaplan-Meier method.

**Figure 2:**
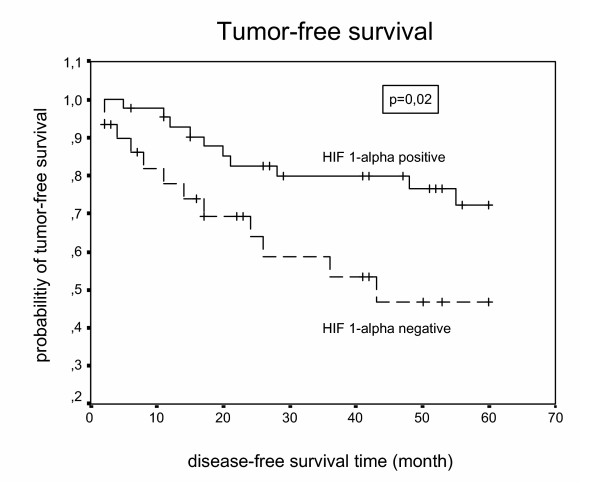
Tumor-free survival of patient with oral squamous cell carcinoma in dependence on HIF-1 expression calculated by the Kaplan-Meier method.

Kaplan-Meier analysis of carcinomas with low HIF 1-α expression (HIF-1α ≤ 5%) showed a significantly poorer disease free (p < 0.01) and overall survival (p < 0.01) (Figs. 1 and 2). No significant association between survival and the expression of CA IX or Glut-1 were found.

**Figure 3:**
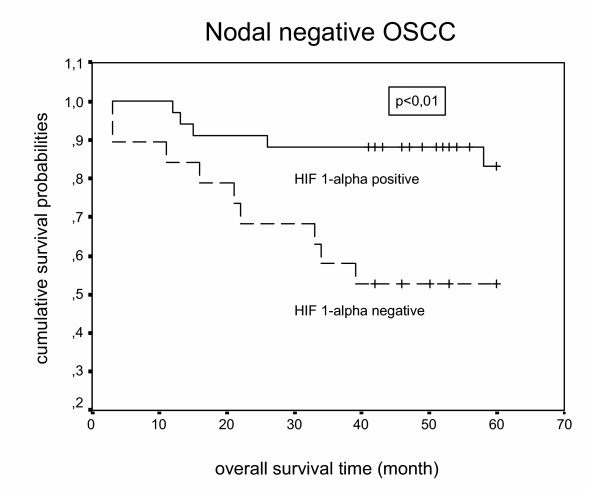
Overall survival of nodal negative patient with oral squamous cell carcinoma in dependence on HIF-1 expression calculated by the Kaplan-Meier method.

In the subgroup node negative T1/T2 tumours of the oral floor low HIF-1α expression was also correlated with a poor overall survival (p < 0.01) (Fig. 3). In multivariate Cox analysis we compared the overall survival and the disease free survival according to clinical usual prognostic factors tumour size and nodal status and also to cyclin D1 and Ki 67 with the HIF-1α expression. Nodal status, tumour size and HIF-1α expression were identified by Cox regression as independent predictors of overall survival (Table 2). Tumour size and HIF-1 expression were also predictors of tumour free survival in multivariate regression (Table 3).

## Discussion

Postsurgical individual therapy decision making in SCC of the oral cavity is hampered by the lack of reliable prognostic markers. Whereas patients with histologically proven lymph node metastases are subjected to radiotherapy, this situation is much more complicated in primary lymph-node negative SCC. Postsurgical radiotherapy in these patients may often be over-treatment, as only a subgroup of poor prognosis lymph-node negative carcinomas would benefit from this type of adjuvant therapy. Nevertheless, at the present state of knowledge no reliable marker exists that identifies these patients.

In recent years it could be shown that the expression of HIF-1α might be a potential candidate for the prognostic assessment for a variety of malignancies. The HIF-1 system represents one of the central cellular anti-hypoxia systems. From a clinical point of view, high levels of HIF 1-α expression seem to predict a poor prognosis for various cancers [17-19].

In our study we aimed to evaluate the prognostic value of HIF 1-α in squamous cell carcinomas of the oral floor with focus on the clinically important subgroup of node negative cases. Up to date only a few data exist about the expression of HIF 1-α in correlation to prognosis in oral SCC, especially after surgical treatment without adjuvant radiotherapy. Unexpectedly, we were able to show that HIF-1α overexpression was related to a significantly poorer 5-year disease free and overall survival. In the clinically most relevant primary node negative T1/T2 tumours, loss of HIF-1α expression identified a subgroup of high-risk patients. A correlation with other established markers could not be defined. We are aware that the use of tissue microarrays might bias immunohistochemical results, especially in SCC's. However, it has to be stressed that the two punches representing one tumour were taken from the centre and the periphery (tumour stromal interface) as previously described by our group for other tumour entities [20]. We have not seen major, statistically relevant differences between the two respective punches. These results are in line with results published by Beasley et al. [4]. Using full sections, this group was able to demonstrate a rather homogeneous distribution of HIF-1 positive cells in SCC's. In detail, no major difference could be found between perinecrotic regions and areas at the tumour-stromal interface. We are therefore strongly convinced that our results are not biased by the use of two tumour punches representing the whole tumour. As we have pointed out ourselves before [21], the TMA technique may have limitations for proteins showing focal expression such as HIF-1α when it is expressed next to small necrotic areas [22]. Nevertheless, even HIF-1α immunohistochemistry on TMAs has provided relevant data in breast cancer [23]. However when there is more diffuse expression of HIF-1α as in oral SCC in the present study, the small tumour cores forming the TMA will more likely be representative.

The literature gives no uniform recommendation for a cut off point of HIF-1α. The used cut off value of HIF-1α expression varies in the literature. Beasley et al. [4] used a cut off value for HIF-1α ≥1% in oral cancer. Other authors used cut off values for HIF-1α between 1% and 5% (Bos R et al. [6] : HIF-1α ≥ 5%.; Bos R et al. [16] : HIF-1α ≥1%). With regard to the variety of the used levels, we choose three different cut off values for HIF-1α expression ≥1%, ≥5% and ≥10%. The 5% threshold of HIF-1α expression discriminated in our investigation two different populations with significant statistical differences in survival prognosis. In this view we have chosen 5% as the cut off value for the main statistical analysis in our study. In regard of the existing literature we also determined the cytoplasmic HIF-1α staining. However, in accordance to Beasley et al. [4] a cytoplasmic staining was a rather rare event and did not influence the statistical results.

In contrast to some adenocarcinomas (e.g. breast cancer [6]), our results indicate that HIF-1α overexpression is related to good prognosis.

The frequent diffuse type of HIF-1α overexpression is in contrast with most adenocarcinomas where usually perinecrotic, hypoxia induced HIF-1α expression is seen [6,24]. In breast cancer, diffuse type of HIF-1α overexpression is probably non-functional [25]. The diffuse pattern of HIF-1α staining is not hypoxia related but is due to alterations in oncogene or tumour suppressor genes (review Semenza [2]). The frequent diffuse HIF-1α expression in SCC of the oral cavity is probably also not hypoxia related. This hypothesis is substantiated by the lack of CAIX and Glut-1 expression, 2 important downstream targets of HIF-1α in the present study. This indicates that the expression of HIF-1α in oral SCC is rather oncogene/tumour suppressor gene related. In view of the observed expression of HIF-1α expression in the higher layers of normal squamous epithelium and the relation to keratinisation in SCC, HIF-1α may have a physiological role in differentiation in cases with diffuse expression. Thereby, HIF-1α expression may be an epiphenomenon rather than constitute a carcinogenetic event in these cases.

Only a small minority of SCC revealed necrotic tumour areas, frequently associated with HIF-1α expression and expression of CAIX and Glut-1, pointing to a hypoxia induced event. These cases tended to be of poor prognosis

Also in adenocarcinomas of the breast, diffuse HIF-1α expression was associated with a better survival than hypoxia induced perinecrotic HIF-1α expression [25].

Our results confirm those by Beasley et al. [4] who also reported a better clinical outcome of HIF-1α overexpressing head&neck SCC, but are at variance with another study on that reported the opposite [26]. Significant variations in the application of postsurgical radiotherapy may however at least in part explain the divergent results.

A small number of cases revealed HIF-1α-expression in fibroblastic cells. Recently, it could be shown that the stromal expression of CA IX indicated an improved patient prognosis in invasive breast cancer. The number oral SCC cases showing stromal HIF-1α expression was too small to evaluate its clinical significance. In view of the potential functional role of HIF-1α expression in the stroma of SCC this deserves to be further studied.

## Conclusion

In summary, we were able to show frequent, probably non-hypoxia related expression of HIF-1-α in oral floor SCC that is related with improved prognosis. Lymph node negative patients lacking HIF-1α may be considered for adjuvant radiotherapy when these results can be confirmed in independent studies.

## Competing interests

The author(s) declare that they have no competing interests.

## Authors' contributions

TF: Project planning, data analysis and writing of the manuscript

RW: Project planning and critical appraisal of the manuscript

PJD: Data analysis and critical appraisal of the manuscript

BB: Project planning, critical appraisal of the manuscript

UJ: critical appraisal of the manuscript

HB: Project planning, data analysis and writing of the manuscript

## Pre-publication history

The pre-publication history for this paper can be accessed here:



## Figures and Tables

**Table 1:** Tumour patient cases in this study

Age (mean)	57 years (range 33 – 87 years)
Sex	83.5 % men, 16,5 % women
Follow-up (mean)	51.8 months (range 4 – 154 months)
T stadium	50 T1 tumours and 35 T2 tumours
N stadium	60 lymph node negative-and 25 lymph node positive carcinoma without extracapsular spread
Grading	17 well differentiated tumours (G1) 59 moderately differentiated tumours (G2) 9 weakly differentiated tumours (G3)

**Table 2:** Multivariate analysis of survival in relation to the HIF-1α expression and other prognostic factors

**Prognosticator**	**P value^a^**	**RR^b^**	**Confidence interval^c^**
Tumor size	0.005	3.4	1.4–7.8
Nodal status	0.003	2.3	1.3–4.2
HIF-1α	0.001	0.2	0.1–0.5
Cyclin D1	0.398	1.3	0.7–2.4
Ki-67	0.602	0.9	0.5–1.5

^a ^The partially nonparametric regression model of Cox (1972) was used to evaluate the predictive power of vatious combinations of prognosticators in a multivariate manner.

^b ^RR, relative risk

^c ^95% confidence interval

**Table 3:** Multivariate analysis of disease free survival in relation to the HIF-1α expression and other prognostic factors

**Prognosticator**	**P value^a^**	**RR^b^**	**Confidence interval^c^**
Tumor size	0.11	2.1	0.8–5.1
Nodal status	0.93	1.0	0.5–2,3
HIF-1α	0.01	0.3	0.1–0.7
Cyclin D1	0.43	1.4	0.6–3,0
Ki-67	0.99	1.0	0.6–1.8

^a ^The partially nonparametric regression model of Cox (1972) was used to evaluate the predictive power of vatious combinations of prognosticators in a multivariate manner.

^b ^RR, relative risk

^c ^95% confidence interval
